# Estimating Photosynthetic Attributes from High-Throughput Canopy Hyperspectral Sensing in Sorghum

**DOI:** 10.34133/2022/9768502

**Published:** 2022-04-08

**Authors:** Xiaoyu Zhi, Sean Reynolds Massey-Reed, Alex Wu, Andries Potgieter, Andrew Borrell, Colleen Hunt, David Jordan, Yan Zhao, Scott Chapman, Graeme Hammer, Barbara George-Jaeggli

**Affiliations:** ^1^The University of Queensland, Queensland Alliance for Agriculture and Food Innovation (QAAFI), Hermitage Research Facility, Warwick, QLD, Australia; ^2^The University of Queensland, Queensland Alliance for Agriculture and Food Innovation (QAAFI), St Lucia, QLD, Australia; ^3^The University of Queensland, Queensland Alliance for Agriculture and Food Innovation (QAAFI), Gatton, QLD, Australia; ^4^Agri-Science Queensland, Department of Agriculture and Fisheries (DAF), Hermitage Research Facility, Warwick, QLD, Australia; ^5^School of Agriculture and Food Sciences, The University of Queensland, Gatton, QLD, Australia

## Abstract

Sorghum, a genetically diverse C_4_ cereal, is an ideal model to study natural variation in photosynthetic capacity. Specific leaf nitrogen (SLN) and leaf mass per leaf area (LMA), as well as, maximal rates of Rubisco carboxylation (*V*_cmax_), phosphoenolpyruvate (PEP) carboxylation (*V*_pmax_), and electron transport (*J*_max_), quantified using a C_4_ photosynthesis model, were evaluated in two field-grown training sets (*n* = 169 plots including 124 genotypes) in 2019 and 2020. Partial least square regression (PLSR) was used to predict *V*_cmax_ (*R*^2^ = 0.83), *V*_pmax_ (*R*^2^ = 0.93), *J*_max_ (*R*^2^ = 0.76), SLN (*R*^2^ = 0.82), and LMA (*R*^2^ = 0.68) from tractor-based hyperspectral sensing. Further assessments of the capability of the PLSR models for *V*_cmax_, *V*_pmax_, *J*_max_, SLN, and LMA were conducted by extrapolating these models to two trials of genome-wide association studies adjacent to the training sets in 2019 (*n* = 875 plots including 650 genotypes) and 2020 (*n* = 912 plots with 634 genotypes). The predicted traits showed medium to high heritability and genome-wide association studies using the predicted values identified four QTL for *V*_cmax_ and two QTL for *J*_max_. Candidate genes within 200 kb of the *V*_cmax_ QTL were involved in nitrogen storage, which is closely associated with Rubisco, while not directly associated with Rubisco activity *per se*. *J*_max_ QTL was enriched for candidate genes involved in electron transport. These outcomes suggest the methods here are of great promise to effectively screen large germplasm collections for enhanced photosynthetic capacity.

## 1. Introduction

Sorghum (*Sorghum bicolor* L. Moench), a C_4_ pathway species and the world's fifth most produced cereal [1], is adapted to a range of environments and retains high photosynthetic efficiency in diverse conditions [2–4]. These characteristics make it a crop of interest for the dual challenge of meeting increasing demands for food and adapting to the effects of climate change [5, 6]. In addition to the C_4_ pathway, which confers adaptation to hot and dry environments, the natural genetic diversity of sorghum provides potential to identify genotypes or genetic loci associated with greater photosynthetic capacity [7]. However, in order to select the photosynthetically favourable genotypes adapted to contrasting environments, tools are required to quantify the biochemical parameters underpinning photosynthetic capacity in a high-throughput manner, removing the phenotyping bottleneck with the traditional gas exchange approach.

Photosynthesis is the process of converting captured solar radiation into chemical energy by fixing carbon dioxide (CO_2_) to form carbohydrates and biomass. Improving photosynthetic capacity is seen as a major target to further improve crop yields [2, 3, 8]. Screening germplasm to directly breed for improved photosynthetic responses to environment conditions is constrained by the complexity of measuring such responses and requires development of higher-throughput indirect phenotyping techniques.

In the C_4_ photosynthetic pathway, the biochemical processes in the mesophyll cells are coordinated with a CO_2_ concentrating mechanism in the bundle-sheath cells [9, 10]. In the mesophyll, CO_2_ is initially fixed by phosphoenolpyruvate (PEP) carboxylase into C_4_ acids, which are then decarboxylated in the bundle sheath cells leading to high CO_2_ levels and hence more efficient carboxylation of Ribulose-1,5-bisphosphate (RuBP) by Ribulose 1,5-bisphosphate carboxylase-oxygenase (Rubisco) [11, 12]. The energy for the regeneration of RuBP in the bundle sheath and PEP in the mesophyll comes from chloroplast electron transport [11]. Due to their key roles in the photosynthetic pathway, the maximal rates of Rubisco carboxylation (*V*_cmax_, *μ*mol m^−2^s^−1^), PEP carboxylation (*V*_pmax_, *μ*mol m^−2^s^−1^), and maximal electron transport rate (*J*_max_, *μ*mol m^−2^s^−1^) largely determine photosynthetic capacity of C_4_ plants and therefore underpin crop productivity. Simulations using a diurnal canopy photosynthesis model predict that canopy growth rate of C_4_ cereals responds largely to changes in *J*_max_ [13]. Quantification of these biochemical parameters is hence of value for selecting enhanced photosynthesis and growth. This is traditionally achieved by conducting gas exchange measurements and fitting observed photosynthetic responses to CO_2_ or light with the Rubisco-activity or electron-transport limited equations in the C_4_ photosynthesis model [11, 14]. However, this method is very time-consuming and not suitable for high-throughput screening of large germplasm collections.

The capacity of leaves to convert absorbed CO_2_ and radiation into biomass also depends on key leaf physiological and structural properties [15]. Two such properties are specific leaf nitrogen (SLN, g m^−2^) and leaf mass per leaf area (LMA, g m^−2^), and both of these are known to be closely associated with photosynthetic capacity [16, 17]. Because nitrogen is a key element in photosynthetic machinery, such as chloroplasts, plant nitrogen status closely links with leaf photosynthetic rates and canopy radiation use efficiency [18–20] and is hence an important parameter in canopy performance modelling [13, 21]. The relationship between leaf nitrogen content and maximal net photosynthesis rate is influenced by LMA which is strongly associated with leaf lifespan and thus affecting the rates of the photosynthetic parameters [15, 16, 22]. However, conventional measurements of SLN and LMA are destructive and slow, limiting their potential to identify germplasm with higher photosynthetic capacity in large breeding programs.

High-throughput plant phenotyping technologies enable the collection of plant biochemical and physiological traits rapidly and nondestructively at large scale [23–26]. Various vegetation indices, which are usually calculated using a few selected wavelengths, have been correlated with plant structural traits (e.g., leaf area index and biomass) or leaf pigment concentration (e.g., chlorophyll). Typical canopy size indicators include normalized difference vegetation index (NDVI) [27, 28] and optimized soil adjusted vegetation index (OSAVI) [29]. Chlorophyll content, on the other hand, has been indicated by indices, such as normalized difference red edge (NDRE) [30] and chlorophyll vegetation index (CVI), which is an indirect measure of nitrogen content [31]. Adjustments to these vegetation indices have also been reported. For example, replacing red bands with red edge when calculating some indices exhibited better performance in estimating chlorophyll content [32].

More recently, hyperspectral imaging sensors with wavelengths in the visible (400-700 nm), near infrared (700-1000 nm), and shortwave infrared (1000-2500 nm) domain have advanced the development of high-resolution spectroscopy techniques. This has led to significant increases in the accuracy and the types of physiological properties that can be retrieved [26, 33]. The linkage between photosynthetic capacity and hyperspectral features therefore constitutes a promising avenue to predict photosynthetic performance of plants across broad scales [20, 34–36]. Various studies have exploited the plethora of bands (>270) and the much narrower band width (<6 nm) available from current hyperspectral sensors to better quantify biochemical and physiological properties in crops [35, 37]. However, most of the studies so far use hyperspectral reflectance to estimate leaf photosynthetic capacity in C_3_ crops [34, 35, 37–41], and similar studies are much rarer for C_4_ crops. At least one study focused on *V*_cmax_, *V*_pmax_, leaf nitrogen content, and specific leaf area from whole spectra reflectance (500-2400 nm) using partial least square regression (PLSR) in C_4_ crop maize [42]. However, *J*_max_ that quantifies the rate of electron-transport limited photosynthetic rate [11] is also important in determining daily biomass growth [13], but has not previously been targeted.

A more comprehensive study on quantifying the key parameters of photosynthesis *V*_cmax_, *V*_pmax_, and *J*_max_ in a C_4_ crop species is proposed. In addition, a high-throughput method to predict key parameters linked to photosynthetic capacity from canopy-level hyperspectral measurements will aid in the selection of genetic material with improved photosynthetic capacity at a large scale. To our knowledge, there are no published previous attempts to estimate the full set of key parameters known to limit C_4_ photosynthesis, at canopy level, using hyperspectral reflectance. Additionally, next generation sequencing techniques have provided a high-throughput and cost-efficient tool for detecting genomic regions associated with crop traits of interest via genome-wide association studies (GWAS) [43–45]. Combining the techniques of hyperspectral sensing and GWAS would greatly facilitate the improvement of photosynthetic capacity and ultimate crop performance, which to date has rarely been explored.

The main objective of this study was to estimate traits associated with photosynthetic capacity from proximal hyperspectral sensing of sorghum canopies. Specifically, we aimed to (i) develop algorithms to predict photosynthetic parameters (*V*_cmax_, *V*_pmax_, and *J*_max_), SLN, and LMA from proximal hyperspectral canopy reflectance captured with a spectrometer attached to a mobile phenotyping platform in two field-grown training sets; (ii) extrapolate the algorithms to GWAS trials grown adjacent to the training sets using a fully genotyped sorghum diversity panel; (iii) evaluate the heritability of the predicted traits; and (iv) undertake GWAS to detect genomic loci associated with the key photosynthetic parameters and identify potential candidate genes to assess the usefulness and robustness of the approaches used in this study.

## 2. Materials and Methods

### 2.1. GWAS Trials

Two field experiments were conducted during two consecutive summer seasons (2019 and 2020) at Gatton Research Station (GAT), Gatton, Queensland, Australia (27°33′S, 152°20′E, 94 m above sea level). GAT1 and GAT2 were sown on 14 January 2019 and 12 November 2019, respectively. Both trials were designed using partial replication with spatially randomised genotypes arranged in rows and columns. There were 875 plots, including 650 genotypes in GAT1, and 912 plots, including 634 genotypes in GAT2, with 70 genotypes in common between trials (Table 1). The genotypes in GAT1 were all inbred lines (*n* = 649) from a sorghum diversity panel comprising world-wide collections [43], and one hybrid was also included. In GAT2, 89% genotypes were hybrids from the Queensland breeding program, and the rest were inbred lines from the sorghum diversity panel. Each plot (4.5 m length and 3 m width) sown to a genotype consisted of four rows. Both trials were planted with a GPS precision planter at a population density of 108,000 plants ha^−1^. For both trials, 150 kg of nitrogen per hectare was applied preplanting, and plots were irrigated regularly to provide nutrient and water nonlimiting conditions. The temperature, photosynthetic photon flux (PPF), and relative humidity (RH) from 6 am to 6 pm for the duration of each trial are shown in Table 1.

### 2.2. Training Sets

Adjacent to each of the GWAS trials, a training set comprising a representative sample of the lines in the GWAS trials was used to collect ground truth data for association with hyperspectral measurements. Completely randomised block designs (row-column) were also used in the training sets. The middle two rows (0.63 m row spacing) of each four-row plot were used for the ground truth data collection while the outside two rows (0.75 m row spacing) were guard rows. The training set in 2019 (TS1) consisted of 80 plots comprising 60 genotypes which were all inbred lines and also included in GAT1. In the training set of 2020 (TS2), there were 108 plots with 93 genotypes of which 63 (68%) were hybrids. There were 19 genotypes in common between TS1 and TS2. Due to differences in germination and vigour of the diverse germplasm used, there was substantial variability in final plant establishment in both trials. The ground truth measurements were only taken from plots which had good establishment, which reduced the number of possible observations that could be used to develop the models. To maximise the number and the range of observations, the ground truth data from TS1 and TS2 were pooled.

### 2.3. Ground Truth Measurements in the Training Sets

In both trials, gas exchange measurements were taken under mostly cloudless conditions (between 9 am and 12 pm) between 35 and 50 days after sowing (DAS)), which was during the active vegetative growth period for all genotypes and hence before the switch to reproductive growth which may introduce physiological and metabolic changes, but after full canopy closure. This period is known to be the most critical period for grain production in sorghum [46]. In total, 75 CO_2_ (ACi) and 75 light (Ai) response curves were collected across TS1 (*n* = 31 plots comprising 29 inbred lines) and TS2 (*n* = 44 plots comprising 30 hybrid and 10 inbred lines) with six inbred lines in common between TS1 and TS2. One plant per plot was randomly selected for gas exchange measurements. The ACi curves were performed on the last or second last fully expanded leaf using a LI-6400 (LI-COR, Inc., Lincoln, Nebraska USA) with a 6400-02B Red/Blue LED light source illuminating a leaf chamber of 6 cm^2^. To measure ACi curves, photosynthetically active radiation (PAR) was set at 1800 *μ*mol photons m^−2^s^−1^, flow rate through the chamber at 500 *μ*mol mol^−1^, and temperature was set to leaf temperature measured at the commencement of each curve. Vapour-pressure deficit (VPD) was generally held at around 3.0 kPa, by adjusting the scrubbing of the incoming air via the desiccant. For each ACi curve, the reference CO_2_ levels were set to the sequences of 200, 100, 50, 250, 400, 650, 800, 1000, 1200, and 1400 ppm, with a duration of 1-5 min for each step. Measurements were made at each CO_2_ supply point when gas exchange had equilibrated, at which point, the coefficient of variation for the CO_2_ concentration differential between the sample and reference analysers was below 1%. The light levels for the Ai curves were set at 2000, 1500, 1000, 500, 250, 120, 60, 30, 15, and 0 *μ*mol m^−2^s^−1^. The other controls were set as follows: reference CO_2_ (constant at 400 *μ*mol mol^−1^), flow (500 *μ*mol mol^−1^), temperature was set to leaf temperatures, and humidity was controlled by scrubbing incoming air to maintain a VPD around 3.0 kPa. The duration for every light level was 1-3 min. Sample and reference analysers were matched before each data point was logged.

A small square section of the leaf (1.6 cm^2^) was collected with a leaf punch from the same leaf section as was used for gas exchange measurements. The leaf sections were dried at 80°C and weighed to calculate LMA (g m^−2^). Percent nitrogen of each sample was determined with a continuous flow isotope ratio mass spectrometer (CF-IRMS), and SLN (g m^−2^) was calculated by multiplying percent nitrogen with LMA. Across the two training sets, 129 SLN and 169 LMA observations (plots) were obtained, involving 124 unique genotypes.

To generate a maximised dataset and enhance robustness of associating the ground truth data taken in a plot with hyperspectral measurements obtained from the same plot, individual plots, rather than genotypes, were considered as an observational unit.

### 2.4. Canopy Hyperspectral Measurements

**Figure 1:**
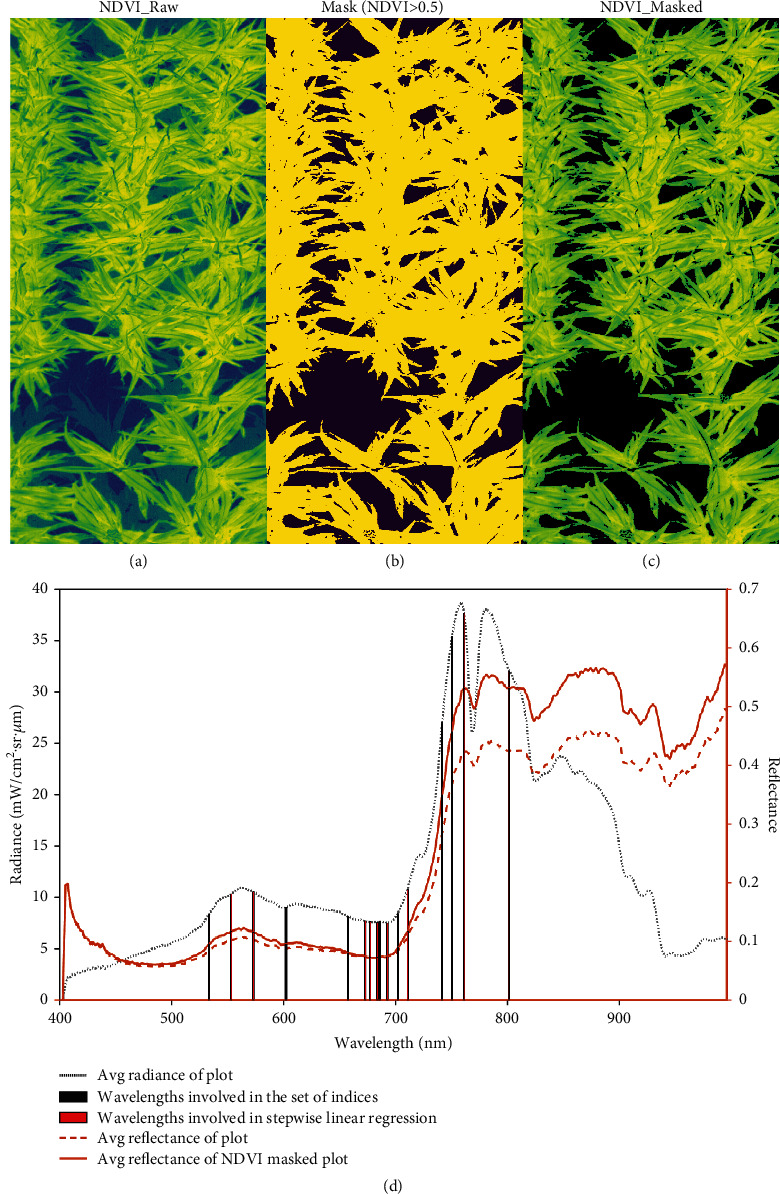
An example (plot 361 in training set 2) of plant canopy area (a) before and (c) after masking by (b) NDVI > 0.5; averaged plot radiance and reflectance before and after masking by NDVI > 0.5 (d).

Hyperspectral data captured before anthesis and around the same time as the ground-truthing data (at 58 and 52 DAS in 2019 and 2020, respectively) was used to associate with the ground truth data. At this stage of sorghum crop growth, canopies are fully closed and nitrogen content of individual leaves is expected to be at a maximum as all mainstem leaves are fully expanded, but, prior to any translocation of nitrogen during senescence [47]. A tractor-based field phenotyping platform (GECKO; developed at The University of Queensland) which enables simultaneous crop canopy proximal sensing was used [48]. The tractor moves at a constant 1.1 metres per second and is integrated with a GPS real-time kinematic system with 2 cm accuracy to locate sampling plots (individual size of 4.5 × 3 m). A microhyperspectral imager (Micro-Hyperspec VNIR model, Headwall Photonics, Fitchburg, MA, USA) mounted on this phenotyping platform (3 m above ground and~1.7 m above the canopy) was used to obtain the spectral response of each pixel (5 × 5 mm) at 272 spectral wavelengths between 395 and 997 nm (visible and near infrared). The resolution was approximately 2.2 nm with 6.0 nm Full Width Half Maxima. A radiometric calibration (dark signal calibration) of the hyperspectral camera was performed weekly. A spectral calibration using the nominal white and spectral diffusers with specific band sets focused on the highest possible spectral resolution was conducted every three months by comparing their respective responses in almost identical illumination conditions. An automated software data calibration pipeline was used to convert raw digital numbers to reflectance values at each pixel. Pixel reflectance was calculated by the ratio between pixel radiance from the microhyperspectral imager and the reference pixel radiance from an upward sensor measuring incoming radiance. To segment plants from soil and remove background noise from lower canopy levels, a threshold of NDVI > 0.5 was applied for each pixel based on the fractional vegetation cover [27, 36, 49], which could ensures only spectral information from green leaves is retained for the reflectance calculations and shadows and other background noise are excluded from the hyperspectral images. After masking by NDVI > 0.5, plant pixels within a plot were averaged to calculate reflectance of each plot. All hyperspectral data was collected from 9 am to 12 pm to minimise the effects of relative orientation of the sun, and no adjustments were made for the sensor or the distribution of leaf angles in the masking. As an example, images, radiance, and reflectance pre- and postmasking by NDVI > 0.5 for plot 361 in 2020 are shown in Figure 1.

A set of hyperspectral vegetation indices known to be associated with photosynthesis was computed from the plot reflectance involving 16 wavelengths as shown in Figure 1. The equations used to calculate the indices in this study were summarised in Table 2.

Note: Wavelengths with black bars show the wavelengths used for calculating the set of vegetation indices known to be associated with photosynthesis; wavelengths with red bars indicate the wavelengths involved in the stepwise linear regression (referring to 2.2).

### 2.5. Determining *V*_cmax_, *V*_pmax_, and *J*_max_ from ACi and Ai Curves

For quantifying the actual photosynthetic parameters, we applied the C_4_ photosynthesis model to the measured ACi and Ai response curves [11, 14]. The CO_2_ assimilation rate (*A*) in the bundle sheath is given by the minimum of either Rubisco carboxylation limited (*A*_*c*_) or electron transport limited (*A*_*j*_) rates:

(1) $$\begin{array}{c} A=\min\left(A_{c},A_{j}\right), \end{array}$$

where,

(2) $$\begin{array}{c} A_{c}=\frac{\left(C_{s}-\gamma *O_{s}\right)V_{\text{cmax}}}{\left(C_{s}+K_{c}\left(1+\left(O_{s}/K_{o}\right)\right)\right)}-R_{d}, \end{array}$$

(3) $$\begin{array}{c} A_{j}=\frac{\left(1-\gamma *O_{s}/C_{s}\right)\left(1-x\right)J_{t}}{3\left(1+7\gamma *O_{s}/\left(3C_{s}\right)\right)}-R_{d}, \end{array}$$

where *O*_*s*_ is the O_2_ partial pressure in the bundle sheath, *γ*∗ is the half of the reciprocal of Rubisco specificity, *K*_*c*_ and *K*_*o*_ are the Michaelis-Menten constant of Rubisco for CO_2_ and O_2_, respectively, and *R*_*d*_ is the mitochondrial respiration rate in the light. All enzymatic constants and variables in the equations above were detailed in a previous study [8].

The *C*_*s*_ (CO_2_ partial pressure in the bundle sheath) is modelled by ambient CO_2_ (*C*_*a*_) entering the leaf via stomata and being diffused into the mesophyll, converted into C_4_ acids then decarboxylated, and released as CO_2_ in the bundle sheath. The supply of CO_2_ to the mesophyll (*C*_*m*_) depends on the intercellular CO_2_ partial pressure (*C*_*i*_), the mesophyll conductance (*g*_*m*_), and the demand term, which is the CO_2_ assimilation rate *A*:

(4) $$\begin{array}{c} C_{m}=C_{a}\times \frac{C_{i}}{C_{a}}-\frac{A}{g_{m}}. \end{array}$$

Here, the effects of the leaf boundary layer and stomatal conductance are incorporated into the *C*_*i*_/*C*_*a*_ term.

The supply of CO_2_ to the bundle sheath (*C*_*s*_) can be limited by enzymatic capacity or chemical energy from the photosynthetic electron transport chain. For the enzyme-limited case, *C*_*s*_ is given by

(5) $$\begin{array}{c} C_{s}=C_{m}+\frac{V_{p}-A-R_{m}}{g_{bs}}, \end{array}$$

where,

(6) $$\begin{array}{c} V_{p}=C_{m}V_{\text{pmax}}/\left(C_{m}+K_{\text{p}}\right), \end{array}$$

where *g*_*bs*_ is the bundle sheath conductance to CO_2_, *R*_*m*_ is the mitochondrial respiration in the mesophyll, and *K*_*p*_ is the Michaelis-Menten constant for CO_2_ associated with PEP carboxylation. Equations (5) and (6) assume carboxylation of CO_2_ by PEP is rate limiting.

The electron transport rate limited CO_2_ supply is given by the same equation structure as in (5), but with the “*V*_*p*_” term replaced:

(7) $$\begin{array}{c} C_{s}=C_{m}+\frac{xJ_{t}/\varphi -A-R_{m}}{g_{bs}}, \end{array}$$

where,

(8) $$\begin{array}{c} J_{t}=\frac{I_{2}+J_{\max}-\sqrt{{\left(I_{2}+J_{\max}\right)}^{2}-4\theta I_{2}J_{\max}}}{2\theta }, \end{array}$$

where *x* is a partitioning factor of electron transport rate between the C_4_ and C_3_ cycles (~0.4) and *φ* is the ATP requirement of the C_4_ cycle (~2 ATP). *I*_2_ is the photosynthetically useful light absorbed by PSII (*PS*_abs_ × incident light) and *θ* is an empirical curvature factor assumed as 0.3 [11].

Equations (3), (4), (7), and (8) were rearranged and fitted to measured Ai curve to infer *J*_max_, *θ*, and *PS*_abs_, which were fed into ACi curve fitting using Equations (2), (4), (5), and (6). Overall, this allows prediction of the Rubisco (*V*_cmax_), PEP (*V*_pmax_), and electron transport (*J*_max_) limited CO_2_ assimilation. The fitting was performed using the numerical solver option in Excel which minimises the sum of square errors of *A* between observed and predicted. The Excel spreadsheet for calculation is shown in Table S1, which shows ACi and Ai fitting with predicted *V*_cmax_, *V*_pmax_, and *J*_max_ for plot 272 in TS2.

### 2.6. Association of Ground Truth Data with Hyperspectral Measurements

#### 2.6.1. Approach 1: Stepwise Multilinear Regression Using the Vegetation Indices

Stepwise regression consists of iteratively adding and removing predictors used in the predictive model, in order to find the subset of variables in the dataset resulting in the best performing model that lowers prediction error. It has been used to select spectral wavelengths highly related to leaf nitrogen, lignin, and cellulose concentrations in diverse species [57, 58]. Stepwise multilinear regression attempts to model the relationship between two or more explanatory variables and a response variable by fitting a linear equation to observed data [59]. Input variables (vegetation indices) are eliminated according to the Pearson correlation coefficient with dependent variables (leaf properties and photosynthetic parameters), which should indicate the most relevant indices to photosynthesis. However, stepwise multilinear regression often suffers from multicollinearity existing in the predictors [58, 60]. In this study, before undertaking stepwise multilinear regression, principal component analysis (PCA) was conducted for the set of hyperspectral vegetation indices in Table 2 to reduce collinearities among them. This resulted in a subset of vegetation indices with reduced correlation between each other which were used in stepwise multilinear regression. The wavelengths used to calculate all the vegetation indices in Table 2 and involved in the subset of vegetation indices are indicated in Figure 1(d). Stepwise multilinear regression using the “MASS” package in R (v 4.0.3) [61] was then conducted to detect the best models for photosynthetic parameters (*V*_cmax_, *V*_pmax_, and *J*_max_) and key leaf properties (SLN and LMA). The best models for each trait were selected, based on Akaike's Information Criteria (AIC) which is commonly used in model selection with lower values indicating a more parsimonious model than a model with a higher AIC [62]. Coefficient of determination (*R*^2^) and root mean squared error (RMSE) were used for model assessment.

#### 2.6.2. Approach 2: Partial Least Square Regression (PLSR) Derived from Spectral Reflectance

In this approach, PLSR was used to correlate the spectra reflectance of all available wavelengths with the photosynthetic parameters (*V*_cmax_, *V*_pmax_, and *J*_max_) and key leaf properties (SLN and LMA) across TS1 and TS2. PLSR has been commonly used in remote sensing spectroscopy to predict plant biochemical and physiological parameters, being able to handle highly correlated predictors and the case of more predictors than observations [60, 63, 64]. The “pls” package in R (v 4.0.3) predicted the traits of interest from reflectance of all the 272 wavelengths, via decomposing the predictor matrix into a set of loadings and scores with the objective of maximising covariance between the scores and response [65, 66]. This process is repeated for a given number of latent variables as the number of loadings and scores necessary to explain sufficient variance in response. The optimal number of latent variables was taken as the minimum number required to minimise the root mean squared error of prediction while not significantly decreasing the cross-validation error, with a maximum of 25 latent variables being considered.

The evaluation of the PLSR models was performed by a leave-one-out cross-validation approach, by training the model on all but one observation and then predicting for the remaining observations [67]. The benefit of many iterations of fitting and evaluating during this cross-validation is that it results in a more robust estimate of model performance as each row of data is given an opportunity to represent the entirety of the test dataset, which is appropriate for a small dataset given the computational cost [68, 69]. This cross-validation approach has been applied in remote sensing of wheat leaf area index, maize and tobacco biochemical traits, crop yield forecasting, and poplar tree photosynthetic capacity predicting from spectral measurements [40, 42, 70–73]. The performances of these regression models were assessed using *R*^2^ and RMSE.

### 2.7. Extrapolating the PLSR Models Built across the Training Sets to the GWAS Trials

To further test the accuracy of the PLSR models built across the training sets, the PLSR models for *V*_cmax_, *V*_pmax_, *J*_max_, SLN, and LMA were used to estimate these traits for each line in the GWAS trials GAT1 and GAT2. Subsequently, GWAS analyses for the two most important photosynthetic parameters (*V*_cmax_ and *J*_max_) in GAT1 were conducted to identify the underlying genetic loci.

#### 2.7.1. BLUPs for the Traits of Interest in the GWAS Trials

To minimise environmental and special effects within trials and perform GWAS, the best linear unbiased predictors (BLUPs) of the predicted traits in the GWAS trials were calculated using a restricted maximum likelihood (REML) by fitting a linear mixed model using the ASReml-R package (Equation (9)) [74, 75].

(9) $$\begin{array}{c} y=X\beta +Zu+\varepsilon , \end{array}$$

where the response vector *y* is modelled by all the fixed effects *β*, random effects *u*, and all the residual effects *ε*. The matrix *X* represents the design matrix for the fixed effects, and the matrix *Z* is the design matrix for the random effects. The fixed effects were composed of main effects for each trial plus any effects associated with linear changes along the rows and columns. The random effects contained sources of error within each trial including replication and any trial specific random row and column effects. The residual effects included trial specific residual effects and first order autoregressive (AR1) effects in both the row and column directions for each trial. The model included genotype as a random effect to predict genotype BLUPs within trials. All possible sources of variation in the BLUPs were allowed for in the linear mixed model [75]. A generalised measure of heritability was calculated due to the complex variance structure, of which the equation is given by (Equation (10)).

(10) $$\begin{array}{c} H^{2}=1-{\bar{SED}}^{2}/\left(2\sigma_{g}^{2}\right), \end{array}$$

where *H*^2^ is the generalised heritability, *σ*_*g*_^2^ represents the genetic variance, and $\bar{SED}$ is the average standard error of difference [76].

#### 2.7.2. GWAS for *V*_cmax_ and *J*_max_ in the GWAS Trial GAT1

All genotypes from the diversity panel used in the GWAS trial GAT1 were resequenced by Diversity Arrays Technology Pty Ltd (http://www.diversityarrays.com). The sequence data was aligned to version v3.1 of the sorghum reference genome sequence [77] to identify SNPs (Single Nucleotide Polymorphisms), resulting in 414,899 SNPs. GWAS analyses were conducted using BLUPs of *V*_cmax_ and *J*_max_ predicted by extrapolating the PLSR models from the training sets to the GWAS trial GAT1. Software FarmCPU [78] was used to conduct GWAS, using 302,631 filtered SNPs (minor allele frequency (MAF) > 0.01). A significant threshold was set as Bonferroni-corrected 0.05/number of effective SNPs [79, 80], resulting in a threshold of *p* value < 1.6e-7.

#### 2.7.3. Pathway Enrichment Analyses Based on Genes within 200 kb from the QTL of *V*_cmax_ and *J*_max_

To further evaluate the reliability of extrapolating the PLSR models for *V*_cmax_ and *J*_max_ from the training sets to the GWAS trials, pathways enriched for genes around the QTL of *V*_cmax_ and *J*_max_ were analysed using *PhytoMine* of *Phytozome* v13 (https://phytozome-next.jgi.doe.gov/phytomine/begin.do), by inputting genes within 200 kb of each QTL detected from the Sorghum_bicolor.Sorghum_bicolor_NCBIv3.47.chr.gff3. Genes identified as enriched in the pathways via *PhytoMine* were defined as candidate genes.

## 3. Results

### 3.1. Variation in Ground Truth *V*_cmax_, *V*_pmax_, *J*_max_, SLN, and LMA across the Two Training Sets

**Figure 2:**
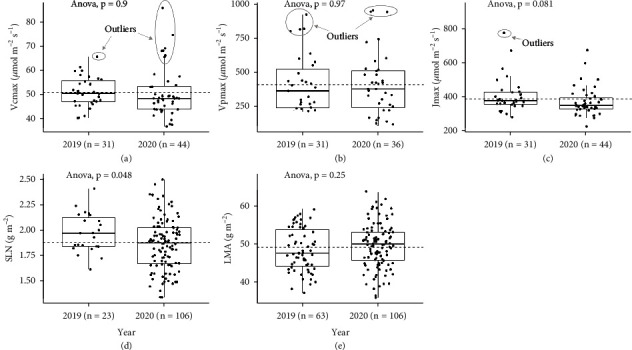
Boxplots showing range of maximal Rubisco carboxylation ((a**)***V*_cmax_), maximal PEP carboxylation ((b) *V*_pmax_), maximal electron transport ((c**)***J*_max_), specific leaf nitrogen ((d) SLN), and leaf mass per area ((e**)** LMA) in training set 1 (2019) and training set 2 (2020).

Substantial variation for all traits measured by ground truthing was observed in the two training sets (Figure 2). In the training set in 2019 (TS1), plot values of *V*_cmax_ had an average of 51.1 *μ*mol m^−2^s^−1^ and ranged from 40.3 to 65.5 *μ*mol m^−2^s^−1^, *V*_pmax_ varied between 123 and 922 *μ*mol m^−2^s^−1^ with a mean of 408 *μ*mol m^−2^s^−1^, and *J*_max_ had an average of 409 with a range of 280 to 773 *μ*mol m^−2^s^−1^. In the training set in 2020 (TS2), *V*_cmax_ varied from 36.8 to 85.6 *μ*mol m^−2^s^−1^ with a mean of 50.9 *μ*mol m^−2^s^−1^, *V*_pmax_ had an average of 410 *μ*mol m^−2^s^−1^ and ranged from 105 to 952 *μ*mol m^−2^s^−1^, and *J*_max_ ranged from 227 to 673 *μ*mol m^−2^s^−1^ with a mean of 383. No significant differences were observed in the photosynthetic parameters between the training sets in two years (ANOVA, *p* > 0.05), and pooled data of observations from individual plots across TS1 and TS2 were used to enrich the results. With the pooled data, a total of 75 ACi and 75 Ai curves were used for fitting *V*_cmax_, *V*_pmax_, and *J*_max_. However, eight ACi curves could not be fitted sensibly with the C_4_ photosynthesis model, possibly due to low data quality caused by high air temperature (> 38°C, Table 1). Given the possible errors from confounding environmental factors in the fittings of *V*_cmax_, *V*_pmax_, and *J*_max_, extreme values (*V*_cmax_ > 65, *V*_pmax_ > 750, and *J*_max_ > 700 *μ*mol m^−2^s^−1^) were treated as outliers and excluded from further analyses as shown in Figures 2(a)–2(c), based on their average values. In total, 67 *V*_cmax_, 60 *V*_pmax_, and 74 *J*_max_ plot observations were effective for further analyses.

SLN varied from 1.6 to 2.4 g m^−2^ with a mean of 2.0 g m^−2^ in TS1 and ranged from 1.3 to 2.5 g m^−2^ with a mean of 1.9 g m^−2^ in TS2 (Figure 2(d)). Pooled data across the two training sets was used for the estimation of SLN (*n* = 129 plots) (Table 1). LMA ranged from 36.0 to 63.5 g m^−2^ (*n* = 169 plots) and did not significantly differ between TS1 and TS2 (Figure 2(e)), and data from the two trials were pooled together. No outliers of SLN or LMA were removed from the following analyses, given no extreme values were observed (Figures 2(d) and 2(e)). Thus, in total, 129 SLN and 169 LMA observations were used for association with hyperspectral data.

### 3.2. Approach 1: Stepwise Multilinear Regression Using the Vegetation Indices

**Figure 3:**
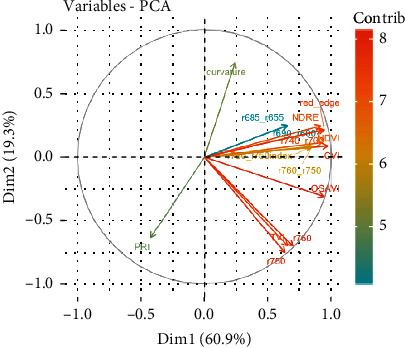
PCA for hyperspectral vegetation indices in Table 1 across two training sets. Note: Length of each arrow represents loading of each variable on dimension 1 and 2; contrib: contribution of each variable to dimension 1 and 2.

The first two components of the PCA captured about 80% of the variation in the set of indices, showing strong collinearities among them (Figure 3). For example, strong correlations were observed among NDRE, Red_edge, and r740_r700, as indicated by large positive loadings on component 1. Similarly, NDVI highly correlated with several indices, such as r760_r750, r760_r750index, and CVI, indicated by large negative loadings on component 1. To reduce the collinearities, a subset of vegetation indices (Red_edge, CVI, OSAVI, r760, curvature, and PRI) was selected as predictors for the traits of interest in the stepwise multilinear regression models, based on the correlations among the indices and their loadings on the first two principal components (Figure 3).

The best models based on the AIC criteria are given in Table 3. All models were significant (*p* < 0.05) for estimating the photosynthetic parameters, despite the low *R*^2^ of around 0.20 (Table 3). The RMSEs for predicting *V*_cmax_, *V*_pmax_, and *J*_max_ were 9%, 35%, and 18% of the mean, respectively, suggesting a modest accuracy in estimations of the photosynthetic parameters from the proximal hyperspectral vegetation indices. Moreover, the vegetation indices detected in the best models for *V*_cmax_, *V*_pmax_, and *J*_max_ were mostly based on near infrared (~800 nm), red edge (~710-750 nm), and green (~550 nm) portions of the spectrum (Figure 1(d)), such as CVI, curvature, and OSAVI, which have previously mostly been used as indicators for variation in nitrogen status and canopy size [28–31, 52]. Interestingly, significant association of *V*_pmax_ and *J*_max_ with an oxygen-A band based index (r760) was observed, which has been used to predict chlorophyll fluorescence [81], suggesting sensitivity of this region to photosynthesis. An indicator of light use efficiency, PRI (based on 531 and 570 nm), showed a high coefficient in the estimators of *V*_cmax_, consistent with the physiological linkages between maximum Rubisco activity and electron transport processes. Red_edge and curvature, known to be sensitive to chlorophyll content [52], were commonly detected in the best stepwise multilinear regression models for SLN and LMA.

### 3.3. Approach 2: PLSR Derived from Reflectance at All Available Wavelengths

**Figure 4:**
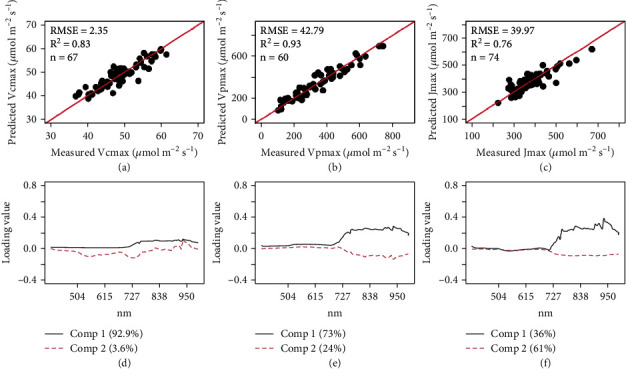
Cross-validated predictions of *V*_cmax_ (a), *V*_pmax_ (b), and *J*_max_ (c) and corresponding loadings with principal components 1 and 2 for *V*_cmax_ (d**)**, *V*_pmax_ (e), *J*_max_**(**f) using partial least square regression (PLSR) and reflectance values at various wavelengths between 395 and 997 nm. Note: Bottom panels (d, e, and f) show model loadings which represent the relative importance of a given spectral wavelength in each model (a, b, and c, respectively); values in brackets indicate the percentage of variance explained by the first two principal components.

Compared with the stepwise multilinear regression models derived from the set of indices, PLSR using reflectance across all the available wavelengths was much more robust for the estimations of *V*_cmax_, *V*_pmax_, and *J*_max_, with *R*^2^ of 0.83, 0.93, and 0.76, respectively (Figures 4(a)–4(c)). The RMSEs for estimating *V*_cmax_, *V*_pmax_, and *J*_max_ were reduced to 4%, 12%, and 10% of the mean, respectively (Figures 4(a)–4(c)). Model loadings, (Figures 4(d)–4(f)) which indicate the contribution of the wavelengths in a specific PLSR model, highlighted the red edge (685-750 nm) and near infrared (a major peak around 950-960 nm) region as important regions for predicting photosynthetic capacity.

**Figure 5:**
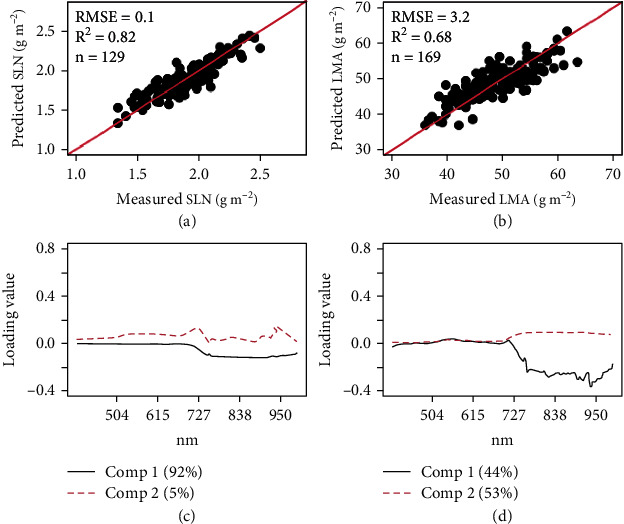
Cross-validated predictions of SLN (a) and LMA (b) and corresponding loadings with principal components 1 and 2 for SLN (c**)** and LMA **(**d) using partial least square regression (PLSR) with reflectance values at different wavelengths between 395 and 997 nm. Note: Bottom panels (c and d) show model loadings which represent the relative importance of a given spectral wavelength in each model (a and b, respectively); values in brackets indicate the percentage of variance explained by the first two principal components.

Using PLSR derived from reflectance of all wavelengths, the predictions of SLN and LMA improved in both *R*^2^ and RMSE compared with the models developed by stepwise multilinear regression using vegetation indices (Figures 5(a) and 5(b)). For SLN and LMA, the RMSE was reduced to 5% and 6% of the mean, respectively. The *R*^2^ reached 0.82 for SLN and 0.68 for LMA. In the models for SLN and LMA, the wavelengths with high loadings largely fell in the near infrared regions with peaks around 722-769 nm and 922-956 nm (Figures 5(c) and 5(d)).

### 3.4. Extrapolating the PLSR Models Built Using the Training Sets to the GWAS Trials

#### 3.4.1. Variation and Heritability of *V*_cmax_, *V*_pmax_, and *J*_max_, and SLN and LMA in GAT1 and GAT2

When using the PLSR models built across the two training sets to estimate the traits in the GWAS trials, reasonable ranges and heritability were observed for all the traits, especially for the two key photosynthetic parameters *V*_cmax_ and *J*_max_ (Table 4). The ranges of the predicted *V*_cmax_ (46-65 *μ*mol m^−2^s^−1^) and *J*_max_ (317-595 *μ*mol m^−2^s^−1^) in GAT1 were particularly comparable with the ground truth measurements in the training sets (Figure 2), suggesting a reasonable accuracy of the extrapolations. This was also supported by the high heritability (around 0.90) of *V*_cmax_ and *J*_max_ in GAT1 (Table 4). The heritabilities of the predictions in GAT2 were lower than in GAT1, because most of the genotypes in GAT2 were hybrids which have less genetic diversity (Tables 1 and 4).

#### 3.4.2. GWAS Based on the Predictions of *V*_cmax_ and *J*_max_ in GAT1

**Figure 6:**
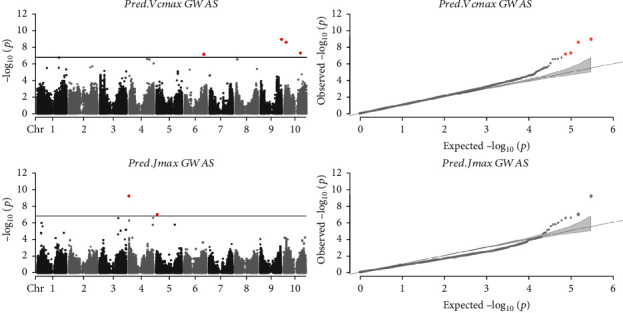
Manhattan and Q-Q plots of GWAS for *V*_cmax_ and *J*_max_ in GAT1. Note: Pred.Vcmax: maximal Rubisco carboxylation rate predicted by the PLSR model for *V*_cmax_ using the pooled training sets; Pred.Jmax: maximal electron transport rate predicted by the PLSR model for *J*_max_ from the pooled training sets; in the Manhattan plots: solid black line showing Bonferroni corrected significant threshold (*p* value <1.6e-7); in both Manhattan and Q-Q plots: SNPs in red passed the significant threshold.

To further evaluate the predictivity of the PLSR models, GWAS analyses were performed on BLUPs of *V*_cmax_ and *J*_max_ predictions in GAT1 (*n* = 649 inbred lines), and given *V*_cmax_ and *J*_max_ have been identified to be the two key photosynthetic parameters for determining net rate of canopy photosynthesis [13]. Four QTL were detected to be associated with the variation in *V*_cmax_ (Figure 6 and Table 5), were located on chromosome 6, 9, and 10, suggesting likely genomic regions associated with the processes of CO_2_ assimilation. In terms of *J*_max_, two QTL located on chromosomes 4 and 5 were identified, providing likely chromosomal regions relevant to the processes of electron transport.

#### 3.4.3. Pathways Enriched for Genes within 200 kb from the QTL of *V*_cmax_ and *J*_max_

To further assess the accuracy of the PLSR models from the training sets, the genes within 200 kb [43] from the QTL detected for *V*_cmax_ and *J*_max_ were analysed by *PhytoMine* (https://phytozome-next.jgi.doe.gov/). One pathway was enriched for five candidate genes of *V*_cmax_, which has been annotated to be associated with UDPG-glucosyl transferase (Table 6). Another pathway, enriched for four candidate genes of *J*_max_, was found to be involved in metabolic processes resulting in the removal or addition of electrons (iron ion binding).

## 4. Discussion

In this study, five key photosynthesis related variables were investigated and predicted from canopy hyperspectral reflectance data, providing an efficient and nondestructive tool to screen genotypes for improved photosynthetic capacity at large scale. Maximal Rubisco carboxylation rate (*V*_cmax_), PEP carboxylation rate (*V*_pmax_), and electron transport rate (*J*_max_), which are the main rate-limiting processes in C_4_-carbon assimilation, were quantified in a diverse set of sorghum genotypes across the two training sets (*n* = 75 plots including 63 genotypes). To date, this is the first attempt to correlate hyperspectral reflectance to detailed fittings of these three parameters from both ACi and Ai curves in C_4_ pathway photosynthesis. The obtained *V*_cmax_ and *V*_pmax_ values were comparable with those reported previously in sorghum [82]. Compared with stepwise multilinear regression, PLSR models improved the prediction accuracy for the three photosynthetic parameters and the other two key leaf properties (SLN and LMA, *n* = 169 plots including 124 genotypes), based on *R*^2^ (~0.80) and RMSE (less than 12% of mean). Subsequently, these PLSR models were extrapolated to two GWAS trials (875 plots with 650 genotypes in GAT1; 912 plots with 634 genotypes in GAT2), with the resulting predictions for both photosynthetic parameters and key leaf properties (SLN and LMA) showing medium to high heritability. Furthermore, the genomic regions associated with *V*_cmax_ and *J*_max_ that were detected by GWAS in GAT1 (*n* = 649 inbred lines) revealed candidate genes involved in the pathways of UDPG-glucosyl transferase and removal or addition of electrons, respectively.

### 4.1. Plot-Based Hyperspectral Reflectance Can Be Used to Predict Leaf Photosynthetic Capacity

#### 4.1.1. Models for *V*_cmax_, *V*_pmax_, and *J*_max_

Hyperspectral reflectance using leaf clips has shown promise for predicting photosynthetic capacity in a variety of plant species [42, 69, 70, 83–85]. However, measurements requiring leaf clips are not practical for screening thousands of breeding lines. To fully achieve high-throughput phenotyping, rather than using handheld spectroradiometers on a leaf-by-leaf basis, estimations of photosynthetic capacity from automated proximal or remote sensing at the canopy level are needed. Apart from greater throughput, canopy measurements also better reflect the whole-plant, which integrates photosynthetic activities measured at the leaf level.

Canopy hyperspectral reflectance has shown promise for estimating *V*_cmax_ and net canopy photosynthetic rate through different approaches, such as airborne-based model inversion in wheat [34]. Another study using canopy hyperspectral reflectance also successfully predicted *V*_cmax_ and *J*_max_ with a ground-based phenotyping platform in tobacco [86]. Moreover, these authors compared three different PLSR approaches including reflectance-based, index-based, and model inversion-based methods, indicating better performance in models based on reflectance and indices than model inversion [86]. A comparison based on leaf- and plot-level PLSR models confirmed the capability of plot-level hyperspectral imaging to predict photosynthetic parameters in transgenic tobacco plants expressing C_4_ photosynthesis pathway genes [40]. In the present study, across 63 sorghum varieties in the training sets, *V*_cmax_, *V*_pmax_, and *J*_max_ were predicted with reasonably high accuracy (*R*^2^ around 0.80 and RMSE within 12% of mean) using PLSR models built from canopy hyperspectral data collected via a proximal phenotyping platform (~1.7 m from canopy). The index-based stepwise multilinear regression models for *V*_cmax_ and *J*_max_ could also estimate the photosynthetic parameters with a reasonably small RMSE around 13% of mean, although with much less percentage of variance explained (*R*^2^ around 0.20). The results from the present study demonstrate the promise of utilising hyperspectral sensing at a canopy level in selective breeding for photosynthetic capacity at large scale and put forward a high-throughput tool to explore genotype by environment interactions of photosynthetic capacity related traits.

#### 4.1.2. Models of SLN and LMA

Nitrogen content has been one of the most successfully predicted traits in crops from both leaf and canopy spectral measurements [20, 87, 88]. In addition to the biochemical parameters, and given the strong associations of nitrogen and LMA with photosynthesis, remote sensing of the key leaf properties has also previously been explored, [16, 89, 90]. Among the estimations from PLSR models in this study, a high coefficient of determination was consistently observed in SLN predictions (*R*^2^ = 0.82), which also had a low RMSE in stepwise multilinear models (10% of mean SLN), demonstrating the effectiveness and suitability of approaches applied in this study.

Another key leaf property, LMA, has been identified as a proxy of photosynthetic capacity in maize [89]. Robust models for predicting LMA from leaf-level hyperspectral reflectance have been reported for wheat and soybean [18, 35, 91]. Additionally, lower RMSE at canopy level than leaf level has been reported for LMA estimations, as multiple scattering in the upper canopy leaf layers could strengthen the expression of key leaf properties in a closed canopy compared with leaf-level measurements [83]. A more recent study in the C_3_ crop zucchini using both leaf- and canopy-level hyperspectral reflectance and PLSR has successfully predicted LMA with *R*^2^ of 0.91 and 0.60, respectively [92]. In the present study, low RMSE (6% of mean LMA) and medium to high *R*^2^ of 0.68 were found in the LMA estimations from canopy hyperspectral reflectance using PLSR. This was also supported by LMA predictions from the stepwise multilinear regression with an acceptable RMSE, 10% of mean. These results indicate that proximally sensed and canopy-based hyperspectral reflectance measurements provide a rapid and robust measure of key leaf properties related to photosynthetic efficiency.

#### 4.1.3. Potential Strategies to Train Robust Models for Predicting Leaf Traits from Canopy-Based Sensing

When using canopy-level hyperspectral data to train leaf-level measurements, shadows, soil background, and canopy structure could be complicating factors that affect the robustness of the model. To address some of the issues with using canopy reflectance, a NDVI > 0.5 mask was applied to each pixel used in the reflectance calculation. This masked out the soil background reflectance and thus minimising the variation in spectral responses from effects associated with canopy heterogeneity (e.g., light or temperature) at the plot level. In addition, some of the noise from canopy structural factors was also minimised in this study by operating within one critical growth stage. However, for future application, developing models suitable for different stages or less sensitive to the variation of canopy structure within a time window would improve utility of the method developed here. Additionally, an automatic thresholding technique (e.g., Otsu) fused with canopy height from LiDAR could be applied in canopy delineation which should be more accurate [93] in delineating the exact canopy areas within a plot. This could reduce spurious reflectance values and thus increase the signal measured from proximal sensing at the canopy level, depending on agricultural contexts (e.g., species or canopy size). Alternatively, combining relevant models that improve the relationships between canopy hyperspectral reflectance and leaf photosynthetic parameters could be useful [34]. Increasing the number of ground truth samples can also improve model performance; however, simply increasing the size of the dataset not only leads to highly complex models but is also affected by the high costs associated with additional measurements, especially in the case of gas exchange measurements which are notoriously slow to obtain [25, 94]. To date, gas exchange measurements are the only realistic measurement of photosynthesis; however, given the confounding factor of variation in photosynthetic capacity within crop canopies of the same genotype [40], this is not ideal.

Reducing the confounding environmental factors (e.g., light or temperature) will also improve model strength when using canopy-based hyperspectral sensing methods to estimate key leaf traits. In this study, all ground truth and sensing data was collected between 9 am and 12 pm, which minimised the effects of sun angle, temperature, and light on canopy reflectance and on photosynthetic rates. Further improvement could be made by incorporating temperature at the time of image capture and tentatively correcting photosynthetic parameters to a standard temperature, as it is one of the most important environmental factors influencing both hyperspectral reflectance and photosynthesis. This was not considered here due to scarce documentation of temperature responses of Vcmax, Vpmax and Jmax in C_4_ crops [39].

### 4.2. PLSR Derived from Entire Wavelength Spectrum Strengthens Model Performance

Compared with the models developed using stepwise multilinear regression, PLSR models were more robust and demonstrated a higher cross validated *R*^2^ and lower RMSE. This is attributed to the fact that additional spectral information was incorporated in the PLSR models using the complete wavelength range compared with the stepwise multilinear regression models [36, 63, 83, 86]. Based on peak loadings (red edge and near infrared), the wavelengths that explained most of the variance in the PLSR models aligned closely with the locations of the wavelength bands selected to develop the best-performing multilinear vegetation index approach. Compared with the published indices that correlate with nitrogen content, a strong overlap was found around the red edge (~710-750 nm) in the present study, consistent with the finding that leaf nitrogen content is linearly correlated with the first derivatives of reflectance at the red edge region around 730 nm [20]. The most important parts of the spectrum for predicting photosynthesis have been shown to be in the visible (400-700 nm) and red edge (710-750 nm) range [83]. In this study, the spectral loadings used to predict photosynthetic parameters had similar peaks to the spectral loadings of SLN and LMA, likely attributed to these features being interdependent [89]. These results provide useful information for selecting relevant wavelengths to predict the traits of interest in further studies.

### 4.3. PLSR Models Built across the Training Sets Can Be Extrapolated to the GWAS Trials

In this study, the PLSR models were extrapolated to the GWAS trials, demonstrating comparable variation and high heritability (~0.80) for the predicted biochemical (*V*_cmax_, *V*_pmax_, and *J*_max_) and key leaf properties (SLN and LMA) in the GWAS trial (GAT1), including predominantly inbred lines. Based on the predictions for these traits in the GWAS trial (GAT2), comprising mostly hybrid lines, relatively lower heritability (~0.5) was observed, as expected, due to similarity among the hybrids both at the molecular and phenotypic level. This suggests hyperspectral sensing is a promising avenue to screen large populations for such traits that have previously been out of reach of crop breeding programs [34, 42, 95]. However, the capacity of green leaves to convert CO_2_ into biomass varies throughout the season mainly due to interactions among genotypes, plant phenological stage and environment [13, 96]. This is likely to further influence predictive skill especially in cases where there is a high within-population variation as a result of the genotype by environment interactions.

The models built across the training sets show sufficient skill to estimate key determinants of photosynthesis in large sorghum mapping populations, grown adjacent to these ground-truth trials, despite potential challenges of predicting leaf photosynthetic capacity from canopy-based hyperspectral sensing. This would not only enable the screening for materials with improved photosynthetic capacity, following identification of genetic loci and potential candidate genes for photosynthetic capacity in the C_4_ crop sorghum but also benefit the quantification of the association between photosynthetic capacity and ultimate biomass improvement in crops. In further applications, it is important to select the best phenology stage for data collection, when the degree of canopy development expressed by leaf area index has more consistent levels of pigment concentration per unit area and more similar spectral response for reducing the impact of such confounding effects associated with plant growth processes (e.g., canopy structure and nitrogen status), [31]. Additionally, further studies to test temporal stability of relationships between canopy reflectance spectra and leaf photosynthetic capacity are needed before extrapolated associations from a specific hyperspectral measurement through the growing season can be made in other crops or agricultural contexts.

Here, GWAS analyses for the two photosynthetic parameters, *V*_cmax_ and *J*_max_, provided useful information for further fine mapping to identify potential candidate genes controlling CO_2_ assimilation and electron transport in sorghum. This is one of the significant and novel outcomes from this study, as this is the first attempt to quantify the genetic basis of the key photosynthetic parameters using hyperspectral sensing in hundreds of lines. Additionally, pathway enrichment analysis for genes within 200 kb from *J*_max_ QTL detected four candidate genes involved in the process of electron transport and light signalling [97]. This means the PLSR model for *J*_max_ built across the training sets was able to capture the genomic loci associated with its phenotypic variation in the sorghum diversity panel. The pathway enriched for genes within 200 kb from the *V*_cmax_ QTL is known to catalyse the transfer of a hexosyl group from one compound to another, as well as function in nitrogen storage [98]. While this is not directly associated with Rubisco activity *per se*, plant nitrogen status is closely associated with Rubisco and leaf photosynthetic rates [18–20]. Additionally, the photosynthetic capacity is colimited by Rubisco activity (*V*_cmax_) and RuBP regeneration, which depends on electron transport (*J*_max_) and the coordination of Calvin cycle enzymes [11, 93]. Enzyme interactions in the Calvin cycle are highly complex [92], and further studies are needed to explore the relevance of the *V*_cmax_ QTL detected here.

## 5. Conclusions

Being able to map crop traits associated with improved resource use efficiency (e.g., nitrogen, light, and water) will contribute to further understanding of the natural variation in photosynthetic processes and enable the exploration of opportunities to modify photosynthesis. This study developed a model using PLSR to estimate maximal Rubisco activities (*V*_cmax_, *R*^2^ = 0.83), maximal PEP activities (*V*_pmax_, *R*^2^ = 0.93), maximal electron transport activities (*J*_max_, *R*^2^ = 0.76), specific leaf nitrogen (SLN, *R*^2^ = 0.82), and leaf mass per leaf area (LMA, *R*^2^ = 0.68) from proximal hyperspectral sensing using two combined training sets (*n* = 169 plots). Further, extrapolating the PLSR models built across the training sets to the GWAS trials including hundreds of lines demonstrates that the predictions of the traits of interest are heritable. GWAS analyses for *J*_max_ in the inbred lines detected genomic regions comprising candidate genes controlling the process of electron transport. While the *V*_cmax_ candidate genes identified here are not associated directly with Rubisco activity *per se*, they are involved in nitrogen storage which is closely associated with Rubisco. These results suggest that the PLSR models from the training sets were able to capture the phenotypic variation in the photosynthetic parameters allowing the discovery of the underlying genetic basis of these important traits.

## Figures and Tables

**Table 1:** Top: mean and maximum daily temperatures, mean daily photosynthetic photon flux, and relative humidity during the two GWAS trials and two training sets in 2019 and 2020; bottom: number of plots and genotypes used in each experiment; and the genotypes in common between trials are in italic.

Year	Temperature (°C)		PPF (*μ*mol s^−1^m^−2^)	RH (%)
	Mean	Maximum	Mean	
2019	26.84	38.98	743.11	62.86
2020	29.22	38.52	1000.95	56.1
Trials	TS1	TS2	GAT1	GAT2
TS1	80 plots (60 genotypes)	19 *genotypes*	60 *genotypes*	36 *genotypes*
TS2		108 plots (93 genotypes)	30 *genotypes*	92 *genotypes*
GAT1			875 plots (650 genotypes)	70 *genotypes*
GAT2				912 plots (634 genotypes)

Note: photosynthetic photon flux (PPF) and relative humidity (RH); the trials in 2019 including the training set TS1 and the GWAS trial GAT1; the trials in 2020 including the training set TS2 and the GWAS trial GAT2.

**Table 2:** Summary of the equations for the set of vegetation indices associated with photosynthesis.

Acronym	Indices	Traits associated	Equations	References
Curvature	Curvature between red and NIR	Chlorophyll content	p683∧2/(p675 × p690)	[50]
CVI	Chlorophyll vegetation index	Chlorophyll content	(p750/p550) × (p670/p550)	[31]
NDRE	Normalized difference red edge	Chlorophyll content	(p750 − p710)/(p750 + p710)	[30]
NDVI	Normalized difference vegetation index	Leaf area index	(p800 − p670)/(p800 + p670)	[28]
PRI	Photochemical reflectance index	Photosynthetic efficiency	(p531 − p570)/(p531 + p570)	[51]
r685_r655		Chlorophyll fluorescence	p685/p655	[52]
r690_r600		Chlorophyll fluorescence	p690/p600	[52]
r740_r700			p740/p700	[52]
r760_r750			p760/p750	[52]
r760_r750index			(*p*760 − *p*750)/(*p*760 + *p*750)	[53]
Red_edge		Chlorophyll content/leaf area index	*p*750/*p*710	[54]
OSAVI	Optimized soil adjusted vegetation index	Leaf area index	(1 + 0.16) × (p800 − p670)/(p800 + p670 + 0.16)	[29]
r750		Vcmax	p750	[34]
r760		Chlorophyll fluorescence	*p*760	[55]
TVI	Transformed vegetation index	Leaf area index	0.5 × (120 × (*p*750 − *p*550)) − 200 × (*p*670 − *p*550))	[56]

**Table 3:** The best models chosen by AIC in stepwise multilinear regression for traits of interest.

Traits of interest	No. observations	Vegetation indices	Coefficients	*R* ^2^	*p* value	RMSE
*V*_cmax_	67	Red_edge	-34.3	0.3	<0.01	4.7
		CVI	2.3			
		PRI	-521.0			
*V*_pmax_	60	OSAVI	-5077.0	0.2	<0.05	143.9
		Curvature	6682.0			
		r760	3899.0			
*J*_max_	74	r760	-1766.0	0.2	<0.05	75.5
SLN	129	Red_edge	0.6	0.2	<0.01	0.2
		OSAVI	-6.1			
		Curvature	-15.8			
LMA	169	Red_edge	30.5	0.2	<0.01	5.0
		CVI	-2.4			
		Curvature	-258.1			

**Table 4:** Range and heritability of predicted SLN, LMA, *V*_cmax_, *V*_pmax_, and *J*_max_ in the GWAS trials.

Site	Trait	Max	Min	Mean	Std.error	*H* ^2^
GAT1	Pred.SLN	2.6	1.4	2.0	0.1	0.85
	Pred.LMA	73.1	50.3	58.8	2.0	0.69
	Pred.Vcmax	64.6	45.7	53.8	1.1	0.87
	Pred.Vpmax	811.4	97.7	399.8	35.3	0.89
	Pred.Jmax	595.2	317.2	457.4	17.4	0.90
GAT2	Pred.SLN	2.1	1.8	1.9	0.1	0.53
	Pred.LMA	65.7	58.8	61.8	1.8	0.52
	Pred.Vcmax	48.6	40.2	43.2	0.9	0.73
	Pred.Vpmax	579.5	258.4	406.8	37.7	0.59
	Pred.Jmax	514.6	443.1	472.9	14.3	0.56

Note: Pred.: predictions for traits in the GWAS trials from the PLSR models built using the pooled training sets; *V*_cmax_ (*μ*mol m^−2^s^−1^): maximal Rubisco carboxylation; *V*_pmax_ (*μ*mol m^−2^s^−1^): maximal PEP carboxylation; *J*_max_ (*μ*mol m^−2^s^−1^): maximal electron transport rate; SLN (g m^−2^): specific leaf nitrogen content: LMA (g m^−2^): leaf mass per area; *H*^2^: generalised heritability.

**Table 5:** QTL identified for *V*_cmax_ and *J*_max_ in GAT1.

Trait	QTL	Chromosome	Position (bp)	*p* value	MAF
Pred.Jmax	qJmax4.1	4	747956	6.86E-10	0.3
Pred.Jmax	qJmax5.1	5	3363160	1.08E-07	0.1
Pred.Vcmax	qVcmax6.1	6	53165713	6.76E-08	0.1
Pred.Vcmax	qVcmax9.1	9	58600798	1.12E-09	0.2
Pred.Vcmax	qVcmax10.1	10	5271782	2.56E-09	0.2
Pred.Vcmax	qVcmax10.2	10	43584867	4.95E-08	0.1

Note: Pred.Vcmax: maximal Rubisco carboxylation rate predicted by the PLSR model for Vcmax using the pooled training sets; Pred.Jmax: maximal electron transport rate predicted by the PLSR model for Jmax from the pooled training sets; Position (bp): the physical positions of QTL identified on the sorghum reference genome v3.1; MAF: minor allele frequency.

**Table 6:** Pathway enrichment analyses for candidate genes within 200 kb from the QTL of *V*_cmax_ and *J*_max_.

Candidate genes	Chr	bp_start	bp_end	Distance to QTL	Closest QTL	Pathway	GO annotation
*Sobic.006G174000*	6	52,994,972	52,996,405	169,308	qVcmax6.1	PWY-2902	UDPG-glucosyl transferase
*Sobic.006G174300*	6	53,001,457	53,004,943	160,770			
*Sobic.006G174400*	6	53,005,433	53,007,221	158,492			
*Sobic.006G174500*	6	53,012,401	53,014,032	151,681			
*Sobic.006G174600*	6	53,021,800	53,023,200	142,513			
*Sobic.004G008300*	4	733,525	734,761	13,195	qJmax4.1	PWY-5129	Iron ion binding, SUR2 and oxidation-reduction process
*Sobic.004G008800*	4	763,419	764,817	15,463			
*Sobic.004G008900*	4	769,869	771,113	21,913			
*Sobic.004G008200*	4	724,142	725,780	22,176			

Note: Chr: chromosome; bp_start: the start point of the gene in the reference genome; bp_end: the end point of the gene in the reference genome; distance to QTL: distance of the gene to the closest QTL in bp; closest QTL: the closest QTL to the candidate gene.

## Data Availability

All phenotypic data used to develop the models presented in this manuscript is available here: https://doi.org/10.48610/acbe0df. Genotypic marker data used for GWAS is available upon request to the corresponding author.

## Supplementary Materials

Supplementary Materials Table S1: to show the Excel spreadsheet for ACi and Ai fitting with predicted *V*_cmax_, *V*_pmax_, and *J*_max_ for plot 272 in TS2.
